# Comparative study of bacterial microfiltration in the implant‐abutment interface, with straight and conical internal connections, in vitro

**DOI:** 10.1002/cre2.439

**Published:** 2021-06-20

**Authors:** Larrucea V. Carlos, Navarro C. Carlos, Larrucea SM. Karina, Boda K. Sunil, Padilla E. Carlos, Lobos G. Olga

**Affiliations:** ^1^ Ex‐Postgrado de Rehabilitación Oral Universidad de Talca Talca Chile; ^2^ Servicio de Cirugía Oral y Maxilofacial del Hospital General Universitario Gregorio Marañon de Madrid Madrid Spain; ^3^ Facultad de Odontología Universidad de Talca Talca Chile; ^4^ MDRCBB‐Minnesota Dental Research Center for Biomaterials and Biomechanics University of Minnesota Minneapolis Minnesota USA; ^5^ Departamento de Microbiología Universidad de Talca Talca Chile

**Keywords:** implants, internal connection, micro CT, microfiltration

## Abstract

**Objective:**

to determine the presence of marginal bacterial microfiltration in the IAI in different implant/abutment systems, in vitro.

**Material and methods:**

Fifty‐six implants from seven different brand names, 4 with cone and 3 with straight connections were used, implant and abutment were connected using the Ncm tightening as indicated by each of the manufacturers and then were sealed. The samples were subjected occlusal load and thermal cycling, a first sample of each group was observed by micro CT and in a second sample (both samples randomly selected) length of connection was measured, while the rest of the samples were mounted on devices according to the bacterial microfiltration model with *Porphyromonas gingivalis*.

**Results:**

Two of the conical connection system groups did not present bacterial microfiltration, one of the three straight connection groups only microfiltered in one sample, while the other two conical as well as the two straight connection samples showed different and important levels of bacterial microfiltration, all groups presented a direct relationship between the implant‐abutment adjustment determined by micro‐CT and bacterial microfiltration levels, not related to the connection length.

Conclusion: Only two conical connection systems presented no bacterial microfiltration.

**What is know**

- In implant rehabilitations, a micro space is created in the implant‐abutment interface (IAI). Previous research has shown that oral microbiota can proliferate in this micro space and affect the peri‐implant tissues, causing inflammation and destruction of alveolar bone. The prevention of microbial leakage through IAI is therefore an important goal in implantology.
- This study simulated conditions such as those which the implant‐abutment system is exposed, using occlusal loading and thermal cycles, following the directives of ISO 14801, these same conditions have been simulated in other studies (CIDR 2018).

**What this study adds**

- The precision degree in the manufacture of the parts involved in the IAI as shown by Micro‐CT added to the shape of the connection (conical) ensure the proper fit and no filtration.
- Under the conditions of this study only two conical connection systems presented no bacterial microfiltration.

## 1 INTRODUCTION

The use of dental implants to treat totally or partially edentulous patients has become an integrated therapeutic modality in dentistry (Ekelund et al., 2003; Hsu et al., 2014; Jorge et al., 2013).

In implant rehabilitations, a micro‐space is created always in the implant‐abutment interface (IAI), depending on the different types of implant systems, this interface presents measurements ranging from 1 to 60 micrometers (Mishra et al., 2017). Other authors found IAI of 2 microns in external hexagon implants with titanium abutment, as opposed to external hexagon implants with a zirconium abutment with significantly larger measurements reaching 26.7 microns.

In addition to the micro‐space, the connection design can influence the bacterial activity, both qualitatively and quantitatively (Canullo et al., 2015; Zipprich et al., 2016), conical additives show superiority over nonconical ones, in terms of gap formation, torque maintenance and abutment stability, so they should present better clinical results (Larrucea et al., 2018).

It has been traditionally accepted that peri‐implant mucositis and periimplantitis are induced by a bacterial biofilm; in fact, the presence of periimplantitis is observed in 10% to 50% of all cases of lost implants after the first year of loading, where microorganisms play an important role (Esposito et al., 1998).

However, a systematic literature search to assess potential mechanical and/or biological complications after implant therapy. Based on all currently available, yet limited, preclinical in vivo and clinical evidence, implantoplasty seems not associated with any remarkable mechanical or biological complications on the short‐ to medium‐term.

(Stavropoulos et al., 2019).

Even so, the identification of the microbiota associated with periimplantitis is crucial to understanding its pathogenesis and the bacteria that could serve as microbial biomarkers for this condition (Emecen‐Huja et al., 2015).

Despite the fact that no bacterial species can be identified as being solely responsible for infection in an implant system, it has been suggested that one of the key periodontal pathogens in the development of periimplantitis may be *Porphyromonas gingivalis*, an strictly anaerobic Gram‐negative bacteria, which is not only responsible for periodontal disease in natural dentition, but is also associated with the destruction of tissue around implants (Cortelli et al., 2013; Salcetti et al., 1997).

In vitro studies have described the potential microbial leakage in IAI under both loaded and non‐loaded conditions (Baggi et al., 2013; Gherlone et al., 2016; Koutouzis et al., 2011; Koutouzis et al., 2014). Although such in vitro studies are only close to biological reality, they can be useful in understanding the dynamics of the IAI and therefore can be useful in improving connection design.

Studies of bacterial microfiltration with inoculation of *Escherichia coli*, in 3 types of conical internal connection, conclude that in all three connection systems bacterial growth was observed at 48 h; and that later they begin to die probably due to the decrease of nutrients (Carcini et al., 2016). Similar results were obtained in another study carried out to evaluate microfiltration with *E. coli*, in conventional internal connection and internal connection with double cone, they observed a lower bacterial microfiltration in the double cone connection at 96 hours of incubation (Gherlone et al., 2016).

Bacterial migration through the IAI has also been correlated with the torque applied between abutment and implant, the micro‐movements of the components during chewing cycles and the adjustment between implant and abutment. Although complete prevention of microbial penetration has not been demonstrated in vitro, internal conical connections have shown better results than internal or external hexagonal connections (Larrucea et al., 2018; Tripodi et al., 2015; Verdugo et al., 2014).

Whatever the pillar‐implant connection used, the size of the microspace in the IAI increases under load, an effect known as pumping, where bacterial leakage increases compared to resting conditions (Koutouzis et al., 2014).

The companies that design and manufacture implants have attempted to reduce such leaks by increasing the precision and stability of articulated parts by mechanizing production techniques. The literature describes how the mismatch between pillar and implant components can reach 66 μm in vertical direction, 10° in rotary direction and 99 μm horizontally, although the exact measurements can vary according to the implant system. The tolerance of some systems can be as low as 5 μm and less than 1° in rotation (Binon, 1996; Binon & Mchugh, 1996).

Several authors have studied the importance of the position, size and geometry of microspace in IAI in marginal bone levels and have demonstrated that bacterial colonization is probably the direct consequence of a poor or ineffective degree of tolerance between the implant and abutment, which increases the size of the microspace. The setting precision can affect the penetration of bacteria, thus establishing a microbiological reservoir. Another aspect to take under consideration is the torque proposed by the manufacturer, to obtain the best possible fit in the geometry of the system. The studies have evaluated different torque values applied to the connection of the prosthetic abutment and the implant and concluded that low values produce a poor connection and increases bacterial leakage (Baggi et al., 2013; Larrucea et al., 2018; Verdugo et al., 2014).

As a way to improve sealing, the use of microcomputer tomography (micro‐CT), which has greatly improved since it was first developed in the 1970s, offers the possibility of three‐dimensional imaging for radiological diagnosis, complementing the visualization of the IAI and the correct connection of the implant‐abutment complex without destroying and/or altering the sample and therefore the morphological characteristics in the IAI (Swain & Xue, 2009).

This is the reason why the prevention of microbial leak in the IAI is a major challenge for the design and manufacture of two‐piece implants, which minimize inflammatory reactions and maximize the stability of the bone (Koutouzis et al., 2011).

Therefore, the aim of this study, was to determine in vitro, the presence of marginal bacterial microfiltration in the abutment‐implant interface using different implant systems.

## 2 METHODOLOGY

This experimental study presented a qualitative approach, with a sample of fifty‐six implants from seven different trademarks, four conical and three straight connections, which were prepared following a previously tested bacterial microfiltration protocol (Larrucea et al., 2018). Ethics approval and informed consent were not required for this in vitro study.

**FIGURE 1:**
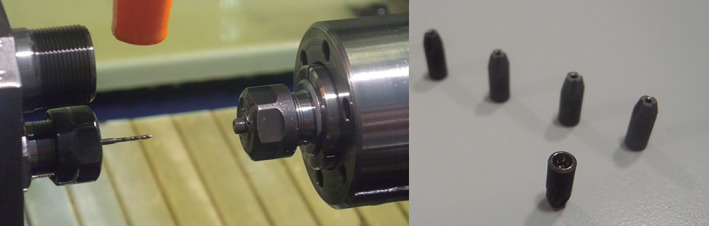
Implant perforation

The apical area of each implant was specially prepared with an orifice of 1 mm diameter following the longest axis of the implant, up to the space where the internal screw that fix the abutment is located (Figure 1).

Each abutment was installed with its respective Ncm to the implant as indicated by the manufacturer and was randomly classified with letters from A to G in order to be blind for the other stages (Table 1).

**TABLE 1:** Experimental groups characteristics and aleatory classification

	Implant				Titanium cementation abutment							
Aleatory group classification	Shape	Implant length (mm)	Implant diameter (mm)	Platform (mm)	Connection type	Connection high (mm)	Antirotating	Gingival high (mm)	Abutment height (mm)	Torque (Ncm)	L dimension (mm)	*n*
A Novel Biocare	Cilinder Conical	10	4.3	4.3	Tri‐channel straight	3.75	Tri‐channel	1	6.5	35	3.749	8
D Biomet 3i	Cilinder Conical	10	4	4.1	Straight Hexag	4	Hexagonal	2	6.5	20	4.040	8
F BioHorizons	Cilinder Conical	10.5	3.8	3.8	Straight Hexag	2	Hexagonal	1.5	7.5	30	1.740	8
B Astra Tech	Cilinder Conical	11	4	4	Morse Cone	2.42	Hexagonal	1.5	6.5	20	2.447	8
E Straumman	Cilinder Conical	10	4.1	4.1	Morse Cone	4.35	Square	2	8	35	4.377	8
C Ticare	Cilinder Conical	10	4.25	4.25	Morse Cone	2.43	Hexagonal	1	6.5	30	2.446	8
G Sweden&Martina	Cilinder Conical	10	4.2	4.5	Morse Cone	3.65	Hexagonal	2	8	20–25	3.642	8

The access to the abutment screw was sealed with Teflon tape and Fermin® (Dental Detax, Ettlingen, Germany). Then the implant was mounted on a standard cylinder, 2.8 cm high and 2.2 cm in diameter, made of transparent self‐curing acrylic resin prepared as indicated by the manufacturer (one third monomer, two thirds polymer), in which the implant was inserted up to the first thread with an inclination of 30° according to ISO 14801: 2008. The preparatory procedure and the random tagging were performed and known by a single investigator.

Perpendicular loading cycles (related to the main axis of the acrylic cylinder) were applied to the samples on the leaning implant abutment, 2000 cycles of 10 kg every 0,5 s.

Then, the 56 samples were subjected to 300 thermal cycles, each cycle consisting of 5 s immersion alternating the water temperature from 5°C to 50°C. The total sample consisted of seven experimental groups, of eight samples each (Table 1).

After loading and thermal cycles, the samples were extracted from the acrylic cylinder and two samples from each group (randomly chosen according to www.random.org) were separated.

**FIGURE 2:**
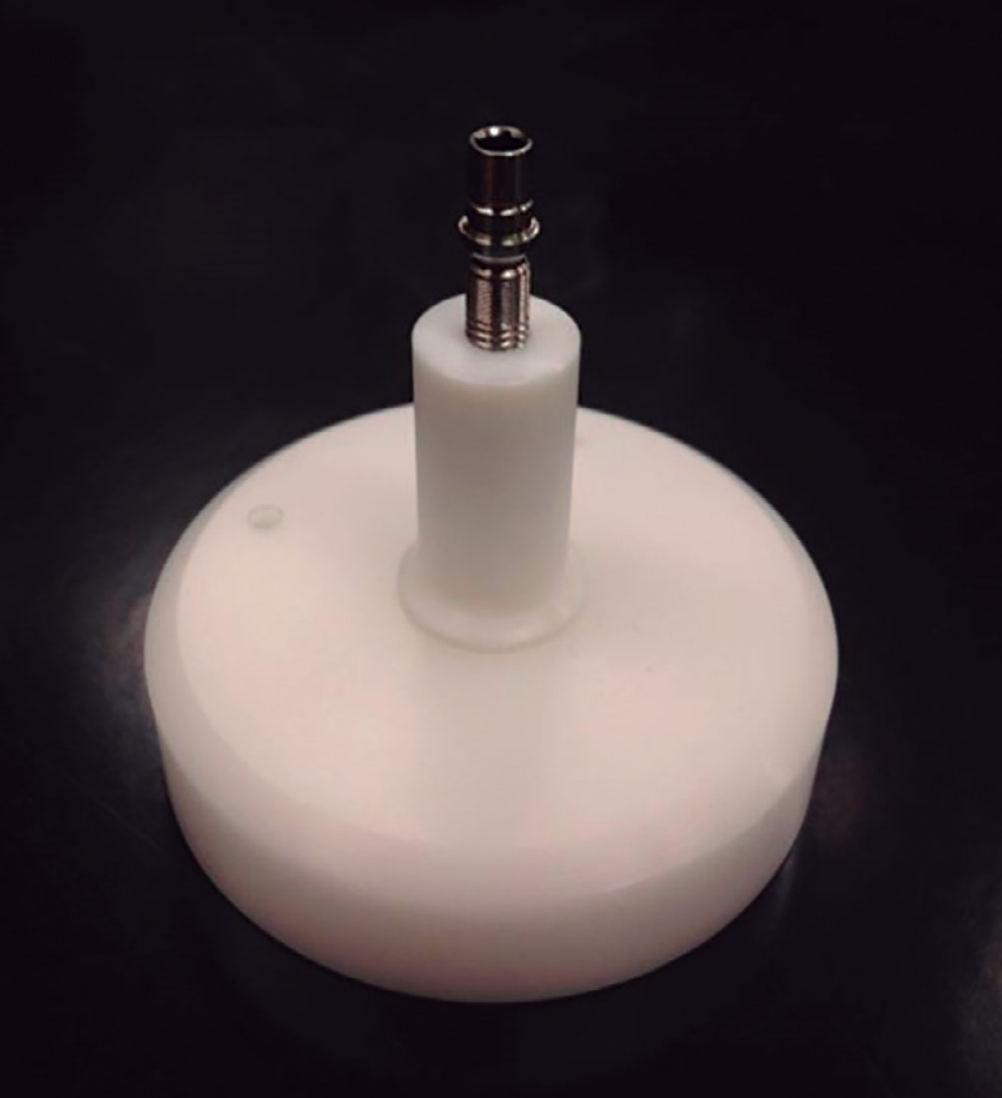
Teflon™ platform with implant mounted for micro‐CT

One sample of each group was set on a platform of Teflon (Figure 2) for the subsequent analysis of micro‐spaces in the IAI by micro CT (Nikon XT H 225, Tokyo, Japan). The X‐ray parameters used during the scanning of the samples were 140 kV tube voltage and a current of 90 μA. For data acquisition, 720 projections were taken with four shots per projection. The 3D reconstruction of the images was carried out using the software CT Pro 3D (Nikon Metrology, Brighton, MI, USA) and the final visualization of the reconstructions by the software VG Studio Max 3.0 (Volume Graphics Gmbh, Heidelberg, Germany). Two independent investigators made the qualitative determination of the presence of micro spaces in the IAI by analyzing the micro TC images.

**FIGURE 3:**
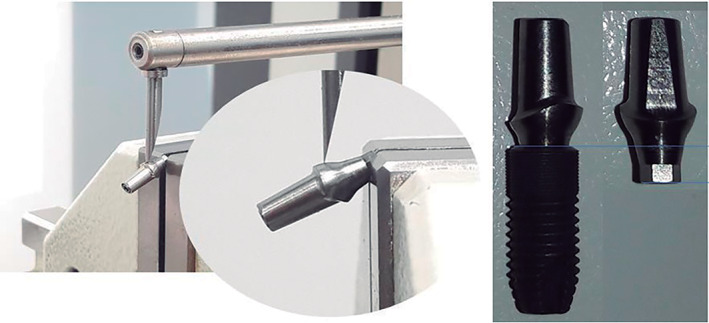
Profilometer, measurement of L dimension

The second sample removed from the group, was used to measure the effective connection, the L dimension, which is the length of the abutment that is inserted inside the implant once it has been screwed. Once insertion level of the abutment into the implant was determined, the abutment was removed to be measured on a Mitutoyo profilometer model CV‐3100, accuracy 125 microns/25 mm (Figure 3), obtaining the L dimension for each sample (Table 1).

**FIGURE 4:**
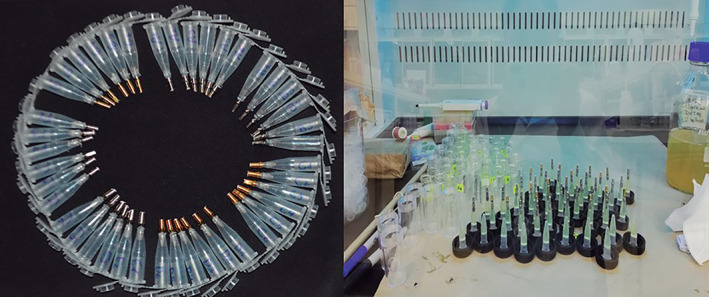
Bacterial leakage model

From the remaining samples (*n* = 42) six samples per group were used to test bacterial microfiltration, one sample from each group was randomly selected to be sealed with cyanoacrylate adhesive (Fenedur, Uruguay) for negative control. The samples were mounted on devices according to the bacterial filtration model (Larrucea et al., 2018; Monardes et al., 2014) modified in Laboratorio de Investigación Microbiológica of Universidad de Talca (Figure 4). Each device was composed by two chambers connected only by the implant; the upper chamber was a 1.5 ml Eppendorf tube (Biologix Research Company, U.S.) with a hermetic cap and the lower chamber was a 10 ml glass tube with a plastic cap. The Eppendorf tube was opened at its lower end and the implant was screwed up to the first thread, sealing the joint with fluid resin (Filtek flow, 3 M ESPE Dental Products St. Paul, Minnesota, U.S.A.). The abutment extending from the tube end was in contact with the lower chamber.

The Eppendorf tube was attached to the plastic cap of the glass tube with cyanoacrylate adhesive (Fenedur, Uruguay), adding a 30G Luer‐type needle (Cranberry=, China), which later would allow the insertion of 5 ml of sterile thioglycollate broth (Becton, Dickinson and Co. 7 Loventon Circle, Sparks, MD 21152USA), and gas release. The device was stored for 24 hours in a sterile atmosphere in a Class II biosecurity cabinet (Nuairetm, Plymouth, U.S.A.) and then sterilized with ethylene oxide. The upper chamber was loaded with a semi‐solid thioglycollate broth of (1.4 ml thioglycollate broth with 0.8% agar) (Becton, Dickinson and Co.). The lower chamber was then loaded with a 50 ul culture of *P. gingivalis* in thioglycollate broth. The bacteria were isolated from clinical origin and identified by molecular biology with the method described by Park et al. (2004). The study began with an inoculum concentration similar to 0.5 Mc Farland (1.5×108 cfu/ml) (Probac do Brasil, Sao Paulo, Brazil), with the implant abutment submerged in the *P. gingivalis* medium.

The samples were incubated for 15 days at 37 °C under anerobiosis conditions in an incubation oven (VWR, Sheldon Manufacturing, Inc. Mod.1510E‐2, U.S.A.). The device was inspected daily by transverse light. Any development of bacteria in the lower chamber was evaluated from the turbidity of the thioglycolate broth, while turbidity in the upper chamber was considered indicative of microfiltration. After determining the microfiltration in the upper chamber, the sample was separated from the uncontaminated. Number of days until microfiltration and sample group were recorded. A sample was taken from the filtrated devices and inoculated in hemina‐menadione blood agar, which was anaerobically incubated at 37 °C for 7–14 days to confirm the growth of *P. gingivalis*, whose identity was confirmed by molecular biology as described above.

## 3 RESULTS

After 15 days since the beginning of the study, the results of the 42 samples in the bacterial microfiltration model are shown in Table 2.

**TABLE 2:** Bacterial leakage by groups and days of incubation

Study group	Subgroup	Incubation (days)															
		1	2	3	4	5	6	7	8	9	10	11	12	13	14	15	
Group A	1	−	−	−	−	−	−	−	−	−	−	−	−	−	−	−	
	2	−	−	+	+	+	+	+	+	+	+	+	+	+	+	+	
	3	−	−	−	−	−	−	−	−	−	−	−	−	−	−	−	
	4	−	−	+	+	+	+	+	+	+	+	+	+	+	+	+	
	**5**	**−**	**−**	**−**	**−**	**−**	**−**	**−**	**−**	**−**	**−**	**−**	**−**	**−**	**−**	**−**	**CN**
	6	−	−	+	+	+	+	+	+	+	+	+	+	+	+	+	
Group B	1	−	−	−	−	−	−	−	−	−	−	−	−	−	−	−	
	**2**	**−**	**−**	**−**	**−**	**−**	**−**	**−**	**−**	**−**	**−**	**−**	**−**	**−**	**−**	**−**	**CN**
	3	−	−	−	−	−	−	−	−	−	−	−	−	−	−	−	
	4	−	−	−	−	−	−	−	−	−	−	−	−	−	−	−	
	5	−	−	−	−	−	−	−	−	−	−	−	−	−	−	−	
	6	−	−	−	−	−	−	−	−	−	+	+	+	+	+	+	
Group C	1	−	−	−	−	−	−	−	−	−	−	−	−	−	−	−	
	2	−	−	−	−	−	−	−	−	−	−	−	−	−	−	−	
	**3**	**−**	**−**	**−**	**−**	**−**	**−**	**−**	**−**	**−**	**−**	**−**	**−**	**−**	**−**	**−**	**CN**
	4	−	−	−	−	−	−	−	−	−	−	−	−	−	−	−	
	5	−	−	−	−	−	−	−	−	−	−	−	−	−	−	−	
	6	−	−	−	−	−	−	−	−	−	−	−	−	−	−	−	
Group D	1	−	−	−	−	−	−	−	−	−	−	−	−	−	−	−	
	2	−	−	+	+	+	+	+	+	+	+	+	+	+	+	+	
	3	−	−	−	−	−	−	−	−	−	−	−	−	−	−	−	
	**4**	**−**	**−**	**−**	**−**	**−**	**−**	**−**	**−**	**−**	**−**	**−**	**−**	**−**	**−**	**−**	**CN**
	5	−	−	−	−	−	−	−	−	−	−	−	−	−	−	−	
	6	−	−	−	−	−	−	−	−	−	−	−	−	−	−	−	
Group E	**1**	**−**	**−**	**−**	**−**	**−**	**−**	**−**	**−**	**−**	**−**	**−**	**−**	**−**	**−**	**−**	**CN**
	2	−	−	+	+	+	+	+	+	+	+	+	+	+	+	+	
	3	−	−	+	+	+	+	+	+	+	+	+	+	+	+	+	
	4	−	−	+	+	+	+	+	+	+	+	+	+	+	+	+	
	5	−	−	+	+	+	+	+	+	+	+	+	+	+	+	+	
	6	−	−	+	+	+	+	+	+	+	+	+	+	+	+	+	
Group F	1	−	−	+	+	+	+	+	+	+	+	+	+	+	+	+	
	**2**	**−**	**−**	**−**	**−**	**−**	**+**	**+**	**+**	**+**	**+**	**+**	**+**	**+**	**+**	**+**	**CN**
	3	−	−	+	+	+	+	+	+	+	+	+	+	+	+	+	
	4	−	−	+	+	+	+	+	+	+	+	+	+	+	+	+	
	5	−	−	−	−	−	+	+	+	+	+	+	+	+	+	+	
	6	−	−	+	+	+	+	+	+	+	+	+	+	+	+	+	
Group G	1	−	−	+	+	+	+	+	+	+	+	+	+	+	+	+	
	2	−	−	+	+	+	+	+	+	+	+	+	+	+	+	+	
	3	−	−	+	+	+	+	+	+	+	+	+	+	+	+	+	
	4	−	−	+	+	+	+	+	+	+	+	+	+	+	+	+	
	5	−	−	+	+	+	+	+	+	+	+	+	+	+	+	+	
	**6**	**−**	**−**	**+**	**+**	**+**	**+**	**+**	**+**	**+**	**+**	**+**	**+**	**+**	**+**	**+**	**CN**

In the samples from Group B and C, both conical connections, there was no bacterial leakage from the lower to the upper chamber of the device, with the exception of a Group B sample which leaked on day 10; Group D had a sample that presented microfiltration on the third day, also on the same day, three of the Group A samples presented bacterial microfiltration; in Group E all its samples with the exception of the negative control leaked on the third day; in Group F, four samples showed microfiltration in day 3 and another two samples on day 6 (including the control sample); all samples from Group G presented microfiltration from day 3 including the control sample.

**FIGURE 5:**
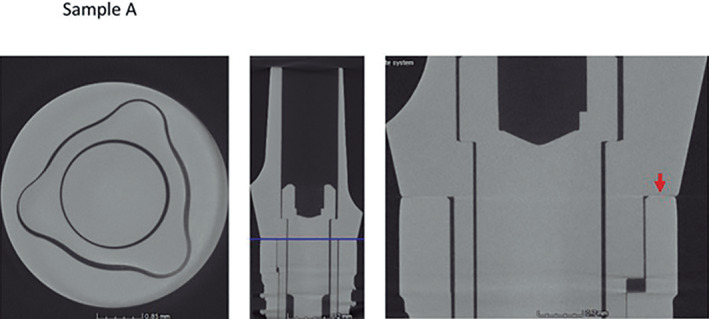
Interface of the abutment of the sample from Group A

**FIGURE 6:**
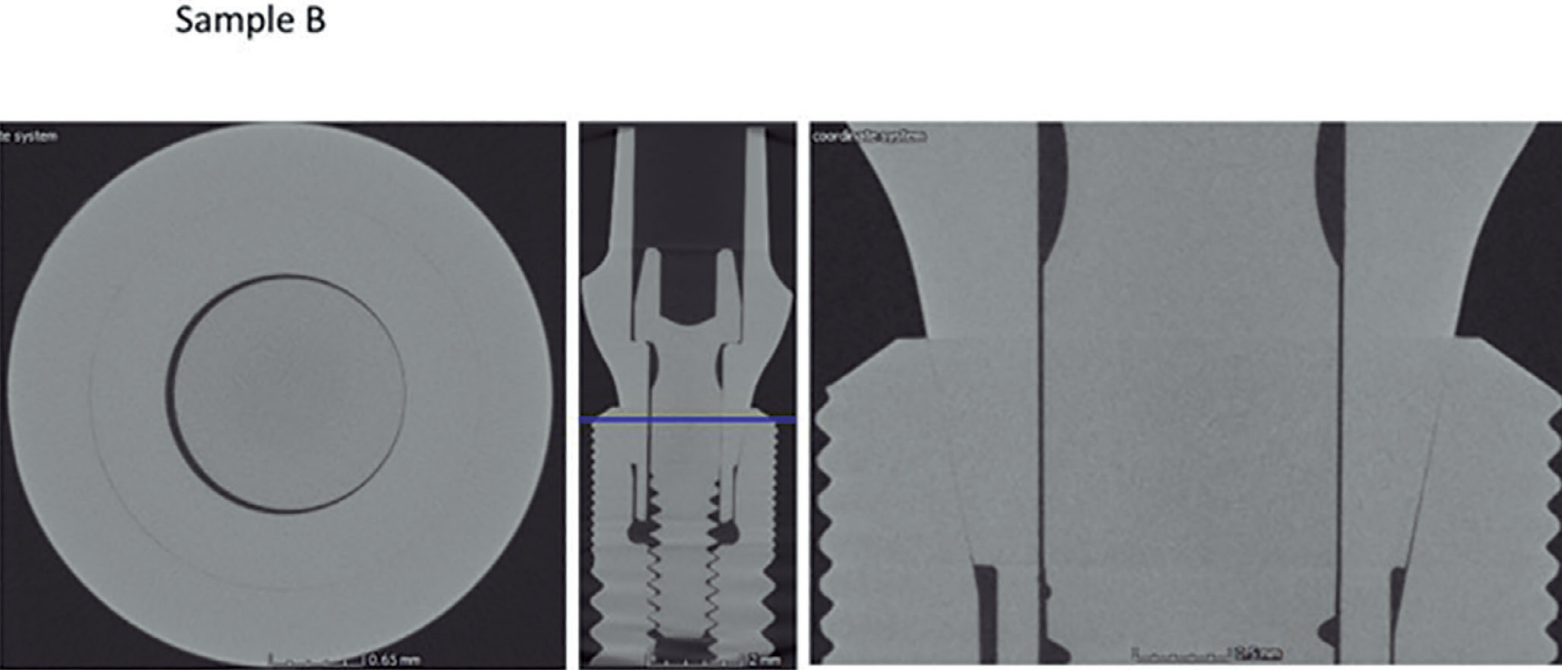
Interface of the abutment of the sample from Group B

**FIGURE 7:**
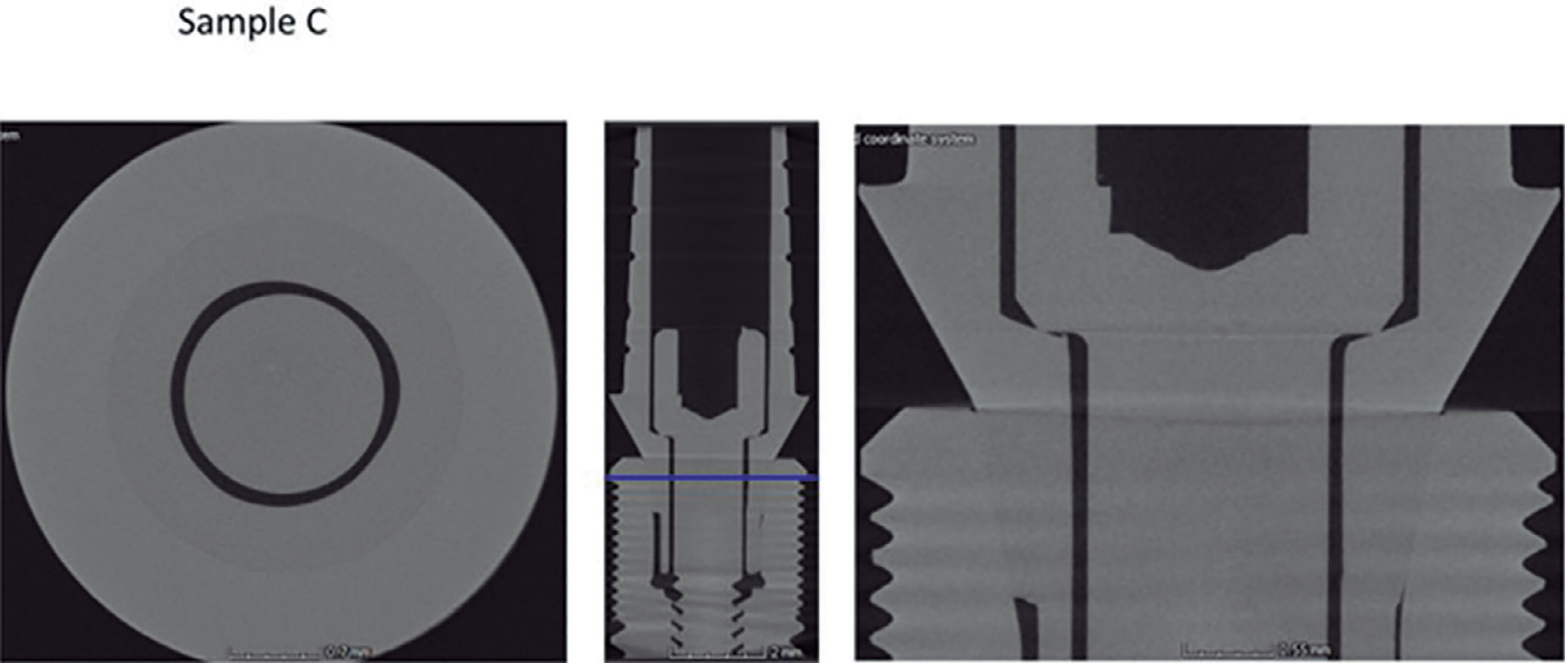
Interface of the abutment of the sample from Group C

**FIGURE 8:**
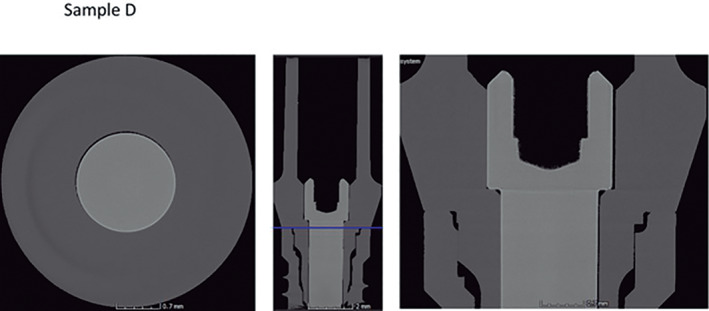
Interface of the abutment of the sample from Group D

**FIGURE 9:**
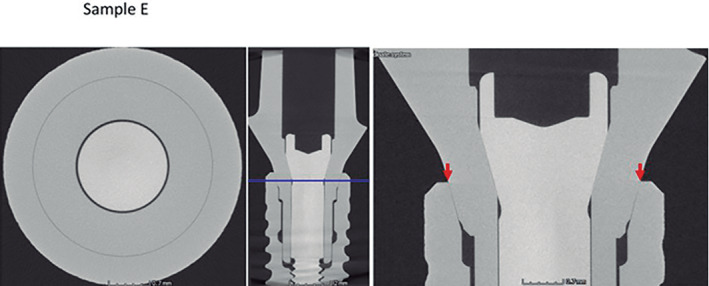
Interface of the abutment of the sample from Group E

**FIGURE 10:**
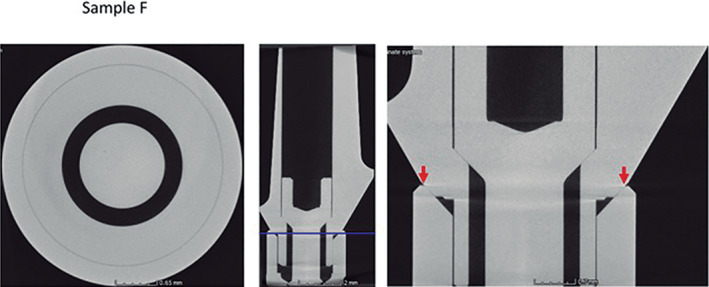
Interface of the abutment of the sample from Group F

**FIGURE 11:**
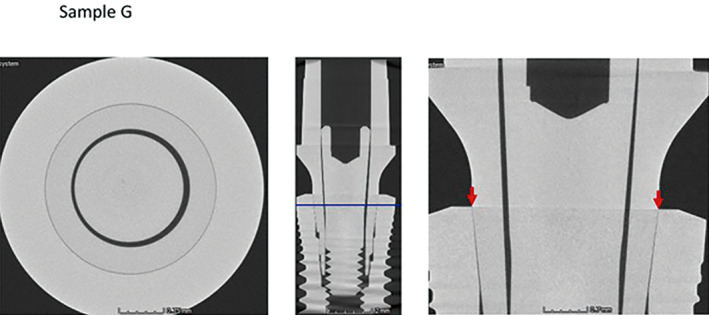
Interface of the abutment of the sample from Group G

Micro CT observation of the frontal and horizontal sections of the different implants (Figures 5, 6, 7, 8, 9, 10, 11) confirmed there where spaces that could result in bacterial microfiltration. Images from groups B and C showed the absence of spaces in the IAI (Figures 6 and 7), images from groups A and D showed a slight lack of adjustment in the IAI (Figures 5 and 8), and the images of groups E, F, and G showed clear spaces in the IAI (Figures 9, 10, 11), so it is possible to assume that the adjustment between the implant and abutment was responsible for the bacterial microfiltration from the lower to the upper chamber of the device.

## 4 DISCUSSION

The bacterial colonization of IAI depends on the adjustment between the implant components, the torque applied to these components and the loading when the implants are in function (Zipprich et al., 2016). This study simulated conditions such as those which the implant‐abutment system is exposed, using occlusal loading and thermal cycles, following the directives of ISO 14801, these same conditions have been simulated in other studies (Baggi et al., 2013; do Nascimento et al., 2012; Koutouzis et al., 2014; Koutouzis et al., 2016; Larrucea et al., 2018; Verdugo et al., 2014).

Under the conditions of this study there was no microfiltration in groups B and C, both with internal conical connections, which correspond to Ticare and Astra Tech implant systems respectively (Table 2), both groups presented perfect adjustment observed by Micro CT (Figures 6, 7) with 2.44 mm length of connection (dimension L). Groups E and G (Strauman and Sweden & Martina) also with internal conical connection, presented bacterial microfiltration through the IAI, this can be related to the lack of adjustment determined by Micro CT (Figures 9, 10, 11), where Group E presented an L dimension of 4.37 mm. and Group G of 3.64 mm. both greater than those presented by groups B and C. With this result we could assume that the L dimension is not related to the bacterial leakage through IAI in the conical connection systems.

All the straight connection implants showed different levels of bacterial filtration in this study, however group D samples (Biomet 3i) was the one with better performance (Table 2) (Figure 8), this could be related to the L dimension of 4.04 mm. which is the largest between straight connections. The other systems studied as Group A (Novel Biocare) although it has an important L dimension of 3.74 mm. also presented filtration (Table 2) and evidence of Gap is observed in its samples (Figure 5), with microfiltration results similar to those of Group F (Biohorizons) who has an internal hexagonal connection of 1.74 mm.

Groups G (conical connection) and F (straight connection), presented early bacterial filtration signs and evidence of poor adjustment between the implant and the abutment, as observed by micro CT, this explains that in both groups the control sample was not able to resist the occlusal and thermal cycles.

Bacterial microfiltration trough interface using this model is a proven method, as demonstrated by Koutouzis et al. (2014), who used *E. coli*; however, in this research we decided to use *P. gingivalis*, as it is commonly isolated in areas with mucositis and periimplantitis (Kumar et al., 2012).

Other authors, such as Baggi et al. (2013), demonstrated that, although the abutment was connected to the implant using the recommended torque wrench prosthetic the geometry of some systems, including internal conical connections, allowed bacteria to enter and exit. When the strength of the connection between the implant and the pillar is lower than recommended, there is a poor connection in the system, as reflected by the presence of bacterial leaks, a situation corroborated by authors such as Baggi et al. (2013), Verdugo et al. (2014) and Larrucea et al. (2018), which is why in this study we only applied the torque recommended by each manufacturer in their respective catalogues.

## 5 CONCLUSIONS

Based on the results obtained in this study, it can be concluded that:

• Only the samples of Groups B and C with conical connection have an adequate adjustment and presented no bacterial filtration.

• The length of the connection does not seem to be fundamental in the abutment‐implant coupling, however in straight connections this length seems to contribute to the sealing.

• The precision degree in the manufacture of the parts involved in the IAI as shown by Micro‐CT added to the shape of the connection (conical) ensure the proper fit and no filtration.

## CONFLICT OF INTEREST

The authors declare no conflict of interest.

## AUTHOR CONTRIBUTION

All the authors have continuously and equally carried out argumentation, writing, laboratory tests and data analysis.

## Data Availability

The data that support the findings of this study are available in Clinical and Experimental Dental Research
